# GM-CSF improves the receptivity of thin endometrium by promoting glandular and stromal cell proliferation in mice and humans

**DOI:** 10.1038/s41420-025-02928-5

**Published:** 2025-12-29

**Authors:** Juan Xie, Qixin Xu, Tao Fu, Ling Zhu, Qianshu Zhu, Xinglin Wang, Qiang Dong, Guoning Huang, Jingyu Li, Zhenshan Yang, Mo Chen, Xiu Luo

**Affiliations:** 1 Chongqing Key Laboratory of Human Embryo Engineering and Precision Medicine, Center for Reproductive Medicine, Chongqing Health Center for Women and Children, Women and Children’s Hospital of Chongqing Medical University, Chongqing, China; 2 Shenzhen Maternity and Child Healthcare Hospital, Southern Medical University, Shenzhen, Guangdong China; 3 College of Veterinary Medicine, South China Agricultural University, Guangzhou, China

**Keywords:** Intrauterine growth, Cell proliferation, Disease model, Experimental models of disease, Outcomes research

## Abstract

Thin endometrium (TE, ≤7 mm) is widely recognized as a critical cause of infertility, recurrent pregnancy losses, and placental abnormalities. Granulocyte-macrophage colony-stimulating factor (GM-CSF) plays a crucial role in tissue repair, but its effect on endometrial regeneration has been less investigated. We employed a thin endometrium mouse model established through unilateral 95% ethanol injury in an animal study and thin endometrium patients in a parallel clinical study. Both mice and patients were randomly apportioned into two groups: the Saline group and the GM-CSF group. We demonstrate that GM-CSF significantly increases endometrium thickness and gland number, promotes the proliferation of stromal cells, and improves the number of embryo implantation sites in the mouse model (*P* < 0.05). GM-CSF significantly (*P* < 0.05) promotes the proliferation of glandular cells, but not stromal cells in humans due to species-specific differential effects. GM-CSF treatment in humans induces upregulation of tissue repair/regeneration genes and enrichment of angiogenesis, cell adhesion, and epithelial proliferation pathways at the transcriptional level. The pregnancy outcomes, implantation rate (24.10% vs. 17.39%), and clinical pregnancy rate (34.78% vs. 26.32%), were both enhanced by GM-CSF compared to the Saline group. The delivery rate shows no statistically significant discrepancy between the two groups. GM-CSF has a positive role in endometrial regeneration and pregnancy outcomes in a thin endometrium. In conclusion, our study provides a novel therapeutic approach for thin endometrium and related infertility.

## Introduction

Embryo implantation initiates maternal-fetal bidirectional crosstalk, which is closely related to endometrial receptivity [1]. The endometrium is a highly regenerative tissue regulated by hormonal fluctuations [2]. The thickness of the endometrium is one of the key factors influencing endometrial receptivity. In ultrasound scanning, an endometrial thickness ≤7 mm compared to the normal uterine cavity is defined as a thin endometrium [3], and insufficient endometrial thickness can lead to pregnancy failure. Physical and biochemical methods that induce endometrial injury could lead to thin endometrium [4].

The incidence of thin endometrium ranged from 5.6% to 37.9% in controlled ovulation cycles, with no distinction observed between drugs and methods of administration in assisted reproductive technologies (ART) [5]. Lower pregnancy and live birth rates, as well as a higher rate of abortion, were found in patients with thin endometrium [6]. In addition, the study on obstetric complications of singleton live birth after fresh in-vitro fertilization (IVF)/ Intracytoplasmic sperm injection (ICSI)- Embryo transfer (ET) treatment found that the incidence rate of hypertensive disorders of pregnancy (HDP) was significantly higher in patients with thin endometrium compared to those with normal endometrium [7]. Deliveries were associated with an overall high rate of main placental-mediated complications and low birth weight in thin endometrium patients, and placentas were characterized by reduced thickness and a high incidence of bilobated placentas [8]. Over the past decades, various therapeutic measures such as hormonal manipulation and drugs, including aspirin, sildenafil citrate, umbilical cord mesenchymal stem cells, and regenerative medicine, have been applied in cases of thin endometrium [9–11]. Despite the wide array of therapeutic interventions, clinical efficacy remains suboptimal, with most regimens yielding only marginal improvements [9]. When the damaged human endometrial stromal cells (ESCs) were co-cultured with Wharton jelly of umbilical cord cells (WJ-MSCs), the proliferation of damaged cells significantly increased, and the percentage of apoptosis decreased [10]. Platelet-rich plasma (PRP) has been reported to enhance endometrial growth and improve pregnancy outcomes in patients with thin endometria [11]. Despite the application of various therapeutic methods in patients with thin endometrium, improvements in promoting endometrial thickness and implantation rates are often limited. Therefore, exploring an effective therapy for thin endometrium remains a challenge.

Granulocyte-macrophage colony-stimulating factor (GM-CSF) is a glycoprotein in the colony-stimulating factor family [12, 13]. It is extensively expressed in the female reproductive system, including the fallopian tubes, endometrium, and ovarian epithelial cells. Furthermore, GM-CSF is regulated by reproductive hormones [14–16]. GM-CSF has been demonstrated to play critical roles in key processes, such as accelerating the development of cultured human embryos in vitro [17]. GM-CSF promoted human blastocyst development in vitro, subsequently increasing the clinical pregnancy and live birth rates [18, 19]. GM-CSF also exhibits beneficial effects in animals. GM-CSF intraperitoneal injection significantly increased endometrial thickness in the thin endometrium mouse model [20]. GM-CSF improved endometrial receptivity through uterine perfusion in the thin endometrium rat model [21]. These studies have shown that GM-CSF is beneficial for tissue repair in various species, regardless of the administration route. However, its role in endometrial regeneration has been less explored. Notably, our bulk RNA-seq data showed that GM-CSF improved repair, regeneration, and epithelial cell proliferation in women with thin endometrium.

In our study, we established a mouse model of thin endometrium to observe endometrial repair following GM-CSF treatment. This study aimed to evaluate the effects of GM-CSF on endometrial regeneration and pregnancy outcomes in both human patients and mice with thin endometrium. The findings may offer new insights for improving clinical treatment strategies and enhancing pregnancy outcomes in individuals with thin endometrium.

## Results

### Construction of a mouse thin uterine endometrial model

**Fig. 1:**
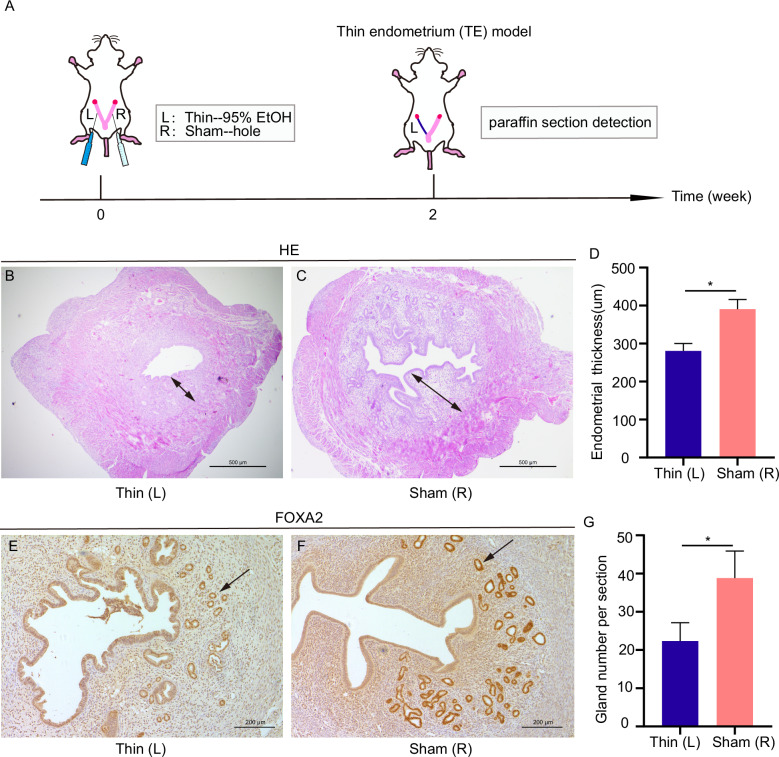
Establishment of a thin endometrium in the mouse model. **A** Schematic diagram of the mouse model establishment and detection methods. **B** H&E staining result of the thin side. **C** H&E staining result of the sham side. Arrows show the thickness of the endometrium. Scale bars, 500 μm. **D** The average endometrial thickness and statistical analysis (±SEM) of the two sides. * indicates *P* < 0.05, *n* = 5. **E** Immunohistochemical FOXA2 expression of the thin side. **F** FOXA2 expression on the sham side. Arrows show the gland in the endometrium. Scale bars, 200 μm. **G** The average gland number per section and statistical analysis (±SEM) of two sides. * indicates *P* < 0.05, *n* = 5.

To test whether GM-CSF plays a role in mouse thin endometrium, we constructed a mouse thin endometrial model (Fig. 1A). Compared to the sham side (the sham-operated side), the endometrial thickness and the number of glands on the thin side (treated with 95% EtOH) were significantly reduced (Fig. 1B). The sham side had a significantly thicker endometrial lining than the thin side (Fig. 1C). Furthermore, the luminal epithelium appeared smooth and continuous on the sham side, whereas it became irregular and jagged on the thin side following ethanol-induced damage.

The endometrial thickness on the modeled side was 280.46 ± 44.59 µm, whereas the endometrial thickness on the control side was 390.71 ± 71.41 µm (Fig. 1D). Likewise, FOXA2 (expressed positively in glands) showed a decrease in the number of glands from 38.80 ± 7.08 to 22.36 ± 9.54 after ethanol exposure (Fig. 1E–G). According to a previous study, a reduction in endometrial thickness and a decrease in the number of glands indicate the successful establishment of our mouse thin endometrium model [20].

### GM-CSF increased endometrial thickness and gland number in mice

**Fig. 2:**
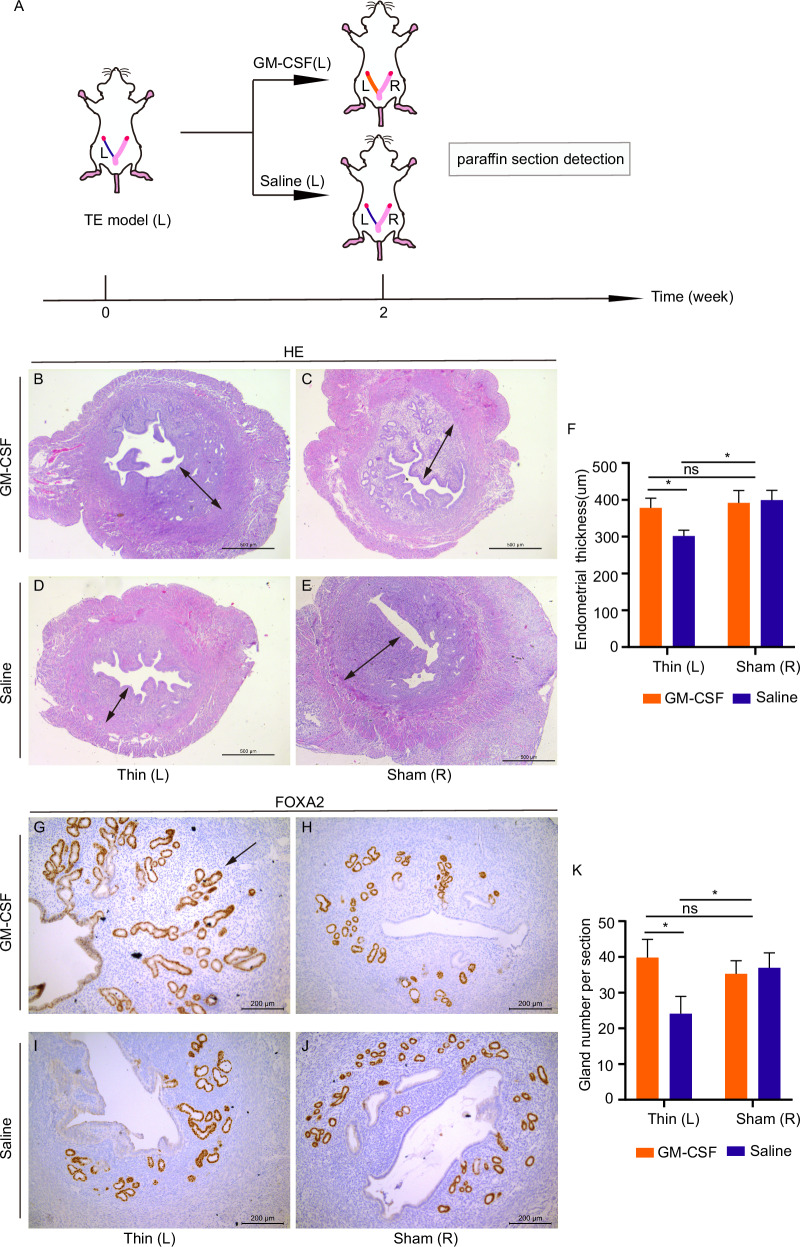
GM-CSF increased endometrium thickness and gland number in mice. **A** Schematic diagram of drug treatment in a mouse model. H&E staining results of the GM-CSF group (**B**, thin side; **C**, sham side) and the Saline group (**D**, thin side; **E**, sham side). Arrows show the thickness of the endometrium. Scale bars, 500 μm. **F** The average endometrial thickness and statistical analysis (±SEM) of the two sides in the two groups. * indicates *P* < 0.05, *n* = 4. FOXA2 immunostaining of GM-CSF group (**G**, thin side; **H**, sham side) and Saline group (**I**, thin side; **J**, sham side). The arrow shows the gland in the endometrium. Scale bars, 200 μm. **K** The number of glands per section in different groups. * indicates *P* < 0.05, *n* = 4.

Following the successful establishment of the thin endometrium mouse model, drug treatments were administered. Endometrial thickness and gland number were assessed two weeks post-treatment (Fig. 2A). The endometrial thickness on the thin side in the GM-CSF group was thicker than that of the Saline group (378.19 ± 58.62 vs. 302.02 ± 34.64 μm, respectively) (Fig. 2B–F). While the endometrium thickness was thinner on the thin side compared to the sham side in the Saline group (302.02 ± 34.64 vs. 399.34 ± 59.96 μm, respectively) (Fig. 2F). When comparing the sham side in the two groups, the thickness was similar (391.83 ± 75.03 vs. 399.34 ± 59.96) (Fig. 2F).

The number of glands increased when treated with GM-CSF compared to the saline group on the thin side, 39.83 ± 19.07 μm in the GM-CSF group, while 24.11 ± 18.09 μm in the Saline group (Fig. 2G–K). Furthermore, the gland number on the thin side was consistent with the sham side in the GM-CSF group (Fig. 2H; 35.26 ± 13.80). The number of glands on the thin side was significantly reduced compared to the sham side in the Saline group (24.11 ± 18.09 vs 36.92 ± 15.76) (Fig. 2K), consistent with the model phenotype. The sham sides between groups showed no significant difference in gland number (36.92 ± 15.76 vs. 35.26 ± 13.80) (Fig. 2K). Taken together, our results show that GM-CSF increased endometrial thickness and the number of glands in the murine endometrium.

### GM-CSF improved endometrial stromal cell proliferation and implantation in mice

**Fig. 3:**
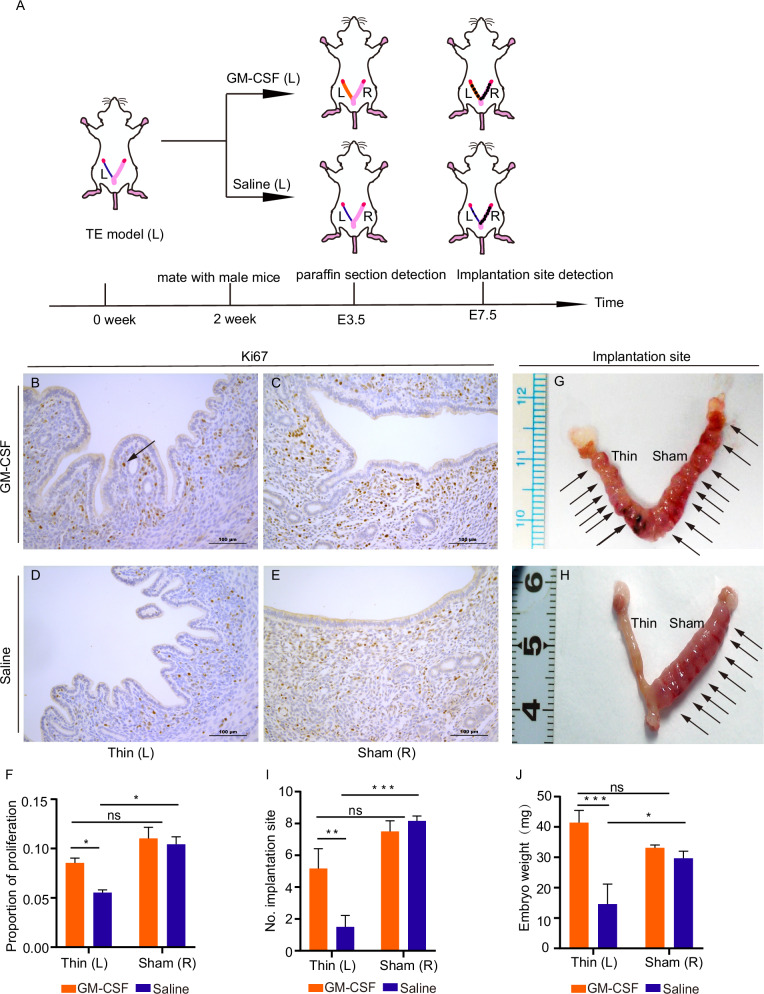
GM-CSF improved proliferation and embryo implantation sites in mice. **A** Schematic diagram of proliferation and embryo implantation sites in the mouse model and detection methods. Immunohistochemical KI67 expression of the GM-CSF group (**B**, thin side; **C**, sham side) and the Saline group (**D**, thin side; **E**, sham side). The arrow shows KI67 staining in stromal cells. Scale bars, 100 μm. **F** The percentage of KI67 in the endometrium. * indicates *P* < 0.05, *n* = 3. The embryo implantation in the GM-CSF group (**G**) and the Saline group (**H**), respectively. Arrows show the embryo implantation sites. **I** Average number of embryo implantation (Mean ± SEM). ** indicates *P* < 0.01, *** indicates *P* < 0.001, *n* = 5. **J** Average weight of embryo implantation (Mean ± SEM). * indicates *P* < 0.05, *** indicates *P* < 0.001, *n* = 5.

We then assessed endometrial proliferation in response to GM-CSF treatment by performing immunohistochemical staining for KI67—a marker of cell proliferation—on uterine sections collected on E3.5 (Fig. 3A). The results revealed that KI67-positive signals were exclusively localized in stromal cells, not in glandular cells. In addition, the percentage of KI67-positive cells was significantly higher in the GM-CSF treatment group than in the Saline group on the thin side (0.11 ± 0.08 vs. 0.06 ± 0.01) (Fig. 3B vs. Fig. 3D). The percentage of KI67-positive cells on the sham side between the two groups showed no significant difference (0.10 ± 0.01 vs. 0.11 ± 0.02) (Fig. 3C, E, F). The percentage of KI67-positive cells on the thin side was lower than the sham side in the Saline group (0.06 ± 0.01 vs. 0.10 ± 0.01) (Fig. 3D vs. Fig. 3E). Our results demonstrated that GM-CSF could promote the proliferation of endometrial stromal cells but not glandular cells in thin endometrium.

We further investigated whether GM-CSF treatment could increase the implantation rate on E7.5. Our results showed that GM-CSF significantly increased the number of embryo implantations on the thin side (5.17 ± 3.06 vs. 1.50 ± 1.76). In contrast, this beneficial effect was not observed on the sham side (8.17 ± 0.75 vs. 7.50 ± 1.64) compared to Saline (Fig. 3G vs. 3H, Fig. 3I). By contrast, the two sides of the saline group showed a significant reduction in the number of embryo implantation sites on the thin side (1.50 ± 1.76 vs. 8.17 ± 0.75) (Fig. 3H). Similarly, we found that GM-CSF could also increase embryo weight on the thin side (41.45 ± 10.53 vs. 14.62 ± 16.03) (Fig. 3J). These results demonstrated that GM-CSF can improve the implantation of mice.

### GM-CSF improved gland number and proliferation in the thin endometrium patients

**Fig. 4:**
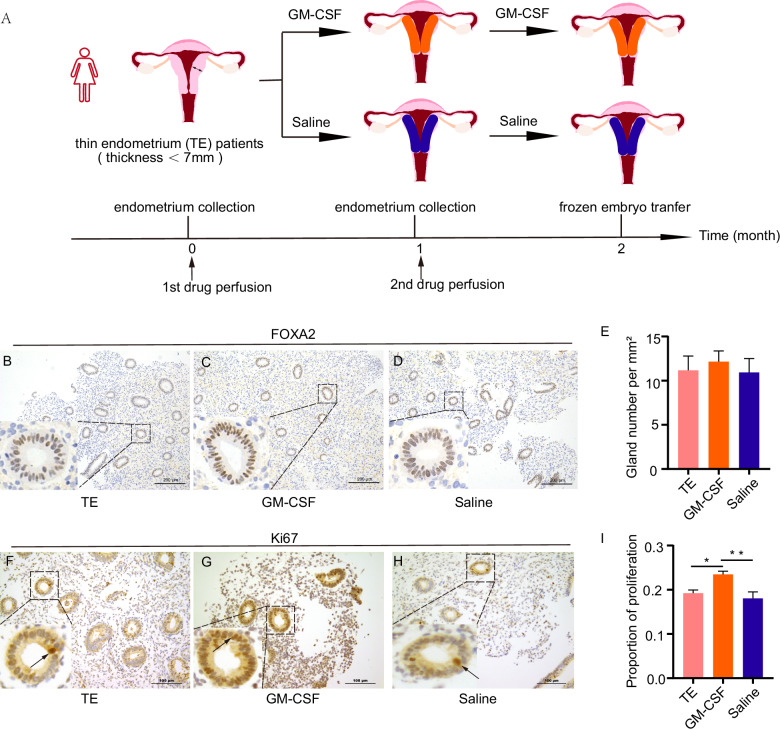
GM-CSF improved gland number and proliferation in humans. **A** The definition of thin endometrium and the process of drug perfusion. FOXA2 immunostain in thin endometrium before drug perfusion (**B**), GM-CSF group (**C**), and Saline group (**D**) after drug perfusion. Scale bars, 200 μm. **E** Average gland number per section in different groups (mean ± SEM). * indicates *P* < 0.05, *n* = 3. **F**–**H** Immunohistochemical KI67 expression of the saline group before saline perfusion. Arrow shows KI67-positive cells in glands. KI67 expression of thin endometrium before drug perfusion (**F**), GM-CSF group (**G**), and Saline group (**H**) after drug perfusion. Scale bars, 100 μm. **I** The percentage of KI67 in the endometrium in different groups. * indicates *P* < 0.05, ** indicates *P* < 0.01, *n* = 3.

The administration method of GM-CSF was shown in Fig. 4A. We found that the gland number was mildly increased after GM-CSF perfusion compared with the untreated group (12.15 ± 2.44 vs. 11.17 ± 3.25) (Fig. 4C, B). The gland number in the Saline group did not change compared with no drug treatment (10.94 ± 3.51 vs. 11.17 ± 3.25) (Fig. 4D, B). However, by quantifying the number of glands in GM-CSF perfusion and saline perfusion in thin endometrial patients, we found that there was no significant variation between the two groups (Fig. 4E).

We then explored whether GM-CSF could affect endometrial proliferation in patients with a thin endometrium. The number of Ki67-positive cells increased in the GM-CSF perfusion group compared to pretreatment levels (0.23 ± 0.02 vs. 0.19 ± 0.01) (Fig. 4G, F). In addition, the number of KI67-positive cells in the GM-CSF group was also increased compared to that in the Saline group (0.23 ± 0.02 vs. 0.18 ± 0.02) (Fig. 4G, H). In contrast, saline perfusion did not induce any notable change in Ki67-positive cell count relative to pretreatment values (Fig. 4I). GM-CSF significantly (*P* < 0.05) promotes the proliferation of human glandular cells, but not stromal cells.

### GM-CSF improved the outcome of the thin endometrium participant

We next investigated whether GM-CSF could improve the outcome of participants with a thin endometrium. To explore this, a total of 84 participants defined as having a thin endometrium (<7 mm) fulfilled all inclusion criteria and were enrolled in this study. Among the 84 participants, 46 women were treated with GM-CSF (treatment group), and 38 were treated with saline (control group). Participants in both groups were similar in demographics and clinical characteristics, as shown in the baseline table (Table 1, *P* > 0.5).

**Table 1:** Baseline patient characteristics in thin endometrium.

	GM-CSF (*n* = 46)	Saline (*n* = 38)	*P*-value
Age (years)	33.20 ± 4.53	33.55 ± 3.19	0.684
Infertility duration (years)	5.00 ± 2.94	6.37 ± 3.82	0.067
BMI (kg/m^2^)	22.79 ± 4.19	21.99 ± 2.46	0.301
AMH (ng/ml)	4.89 ± 3.81	3.74 ± 2.26	0.108
Basal FSH (IU/L)	5.55 ± 1.72	5.47 ± 1.74	0.843
Basal LH (IU/L)	3.56 ± 1.75	3.45 ± 1.92	0.774
Basal E2 (IU/L)	34.96 ± 1.75	34.24 ± 13.30	0.837
Basal PGR (IU/L)	0.27 ± 0.15	0.31 ± 0.16	0.201
Previous IVF failure (n)	1.85 ± 1.05	1.45 ± 1.25	0.114
Case of infertility			
Tubal factor	23 (50%)	23 (60.53%)	0.335
Other female factors	5 (10.87%)	4 (10.53%)	0.960
Mixed factors	18 (39.13%)	10 (26.32%)	0.215
Unknown factors	0	1 (2.63%)	0.268

Values are given as mean ± SD or number (percentage).

*BMI* body mass index, *AMH* anti-Müllerian hormone, *FSH* follicle-stimulating hormone, *LH* luteinizing hormone, *E2* estradiol, *PGR* progesterone receptor, *IVF* in vitro fertilization, *GM-CSF* granulocyte-macrophage colony-stimulating factor.

Pregnancy outcomes are summarized in Table 2. The implantation rate was 24.10% (20 out of 83) for the GM-CSF group and 17.39% (12 out of 69) for the Saline group. Patients who were treated with GM-CSF had a higher clinical pregnancy rate (34.78% vs. 26.32%; *P* = 0.404) compared to patients in the Saline group. In those clinically pregnant patients, the administration of GM-CSF was associated with a higher rate of singleton pregnancy compared to Saline (28.26% vs. 23.68%; *P* = 0.547). However, the delivery rate showed no significant difference between the two groups (68.75%, 11/16 vs. 90.00%, 9/10; *P* = 0.211).

**Table 2:** Profile of pregnancy outcomes.

	GM-CSF (*n* = 46)	Saline (*n* = 38)	*P*-value
Embryo transfer (n)	83	69	-
Implantation rate (%)	20/83 (24.10%)	12/69 (17.39%)	0.312
Clinical pregnancy rate	16/46 (34.78%)	10/38 (26.32%)	0.404
Singleton pregnancy rate	13/46 (28.26%)	9/38 (23.68%)	0.547
Multiple pregnancy rate	3/46 (6.52%)	1/38 (2.63%)	0.547
Abortion rate	5/16 (31.25%)	1/10 (10.00%)	0.211
Delivery rate	11/16 (68.75%)	9/10 (90.00%)	0.211

Values are given as a number (percentage).

Abortion rate indicates the number of pregnant women giving misbirth/clinical pregnancy.

Delivery rate shows the number of pregnant women giving live birth / clinical pregnancy.

Next, we compared the endometrium thickness between GM-CSF-treated and untreated thin endometrial patients. Both groups exhibited similar endometrial thickness in the previous cycle (GM-CSF: 5.83 ± 0.71 mm; Saline: 5.83 ± 0.82 mm). However, endometrium thickness in both groups increased after the drug infusion (6.09 ± 0.76 mm vs. 6.08 ± 0.79 mm; *P* = 0.962) (Table 3).

**Table 3:** Endometrium thickness in the previous cycle and the next implanted cycle.

	GM-CSF (*n* = 46)	Saline (*n* = 38)	*P*-value
Endometrium thickness in previous cycle (mm)	5.83 ± 0.71	5.83 ± 0.82	0.986
Endometrium thickness in the implanted cycle (mm)	6.09 ± 0.76	6.08 ± 0.79	0.962

Values are given as mean ± SD by group.

Additionally, we analyze the embryos transferred on D3 and D5 to explore the influence of the time of embryo culture (Supplementary Tables 1–6). It is interesting to note that embryos transferred on D5 have better delivery rates and endometrial thickness compared to those transferred on D3. The endometrium thickness was 6.25 ± 0.77 mm in the GM-CSF group and 6.23 ± 1.17 mm in the Saline group on D5, and 6.03 ± 0.73 mm vs. 6.00 ± 0.50 mm in the Saline group on D3 (Tables S6 and S3). The delivery rate was 100% (5 out of 5) compared to 80% (4 out of 5) for GM-CSF and Saline administration on D5, but 50% (5 out of 10) versus 100% (5 out of 5) on D3, respectively (Tables S5 and S3).

### The differential expression gene of GM-CSF in the thin endometrium

**Fig. 5:**
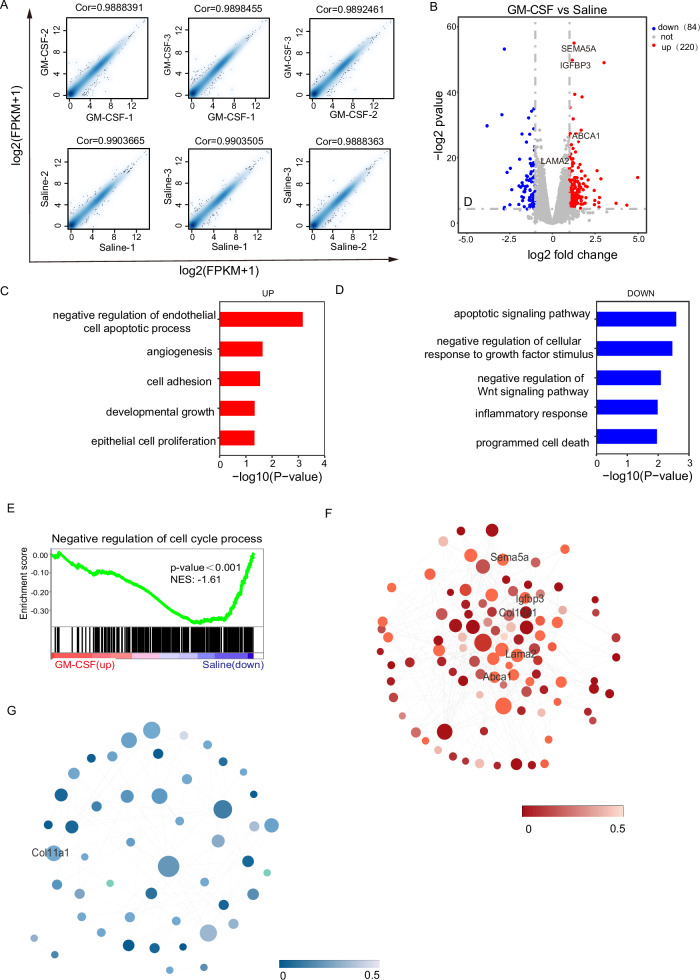
Analysis of RNA-seq differentially expressed genes and GSEA in patients with different treatments. **A** The correlation between the GM-CSF and Saline groups, the Spearman correlation coefficients are also shown. **B** Volcano plot shows 220 upregulated and 84 downregulated differentially expressed genes (DEGs) (*p* < 0.05 and log2 fold change | > 1) in endometrium samples from GM-CSF (*n* = 3) and Saline (*n* = 3) groups. **C**, **D** Significantly enriched GO terms in the biological process (BP) category associated with up (red) and downregulated (blue) mRNAs in the GM-CSF group, respectively. **E** GSEA enrichment in the negative regulation of the cell cycle process. **F**, **G** Network diagram of differentially expressed genes and endometrium-related genes (red represents upregulated genes, and blue represents downregulated genes).

To explore the mechanism of GM-CSF on the thin endometrium, we collected endometrial tissues for RNA-seq. Initially, we observed a strong correlation within the GM-CSF group and the Saline group (Fig. 5A). We identified 304 differentially expressed genes (DEGs) with statistical significance. Among these, 220 genes were upregulated, and 84 genes were downregulated in the GM-CSF group (Fig. 5B). The endometrium repair and regeneration genes, such as SEMA5A, IGFBP-3, ABCA1, and LAMA2, were upregulated in the GM-CSF perfusion group (Fig. 5B). SEMA5A, Semaphorin 5A, works as a new complementary marker for endometrial cancer [22]. IGFBP-3 is a member of the insulin-like growth factor binding protein (IGFBP) family, and IGFBP-3 signaling has been shown to facilitate trophoblast invasion [23]. ABCA1, ATP-binding cassette subfamily A member 1, is involved in fighting intrauterine infections [24]. LAMA2 mediates cell attachment, migration, and tissue invasion during embryonic development [25].

The gene ontology (GO) analysis results revealed that GM-CSF played a positive role in angiogenesis, cell adhesion, and epithelial cell proliferation. Conversely, it suppressed processes such as apoptotic signaling, inflammatory response, programmed cell death, and potently activated the Wnt signaling pathway (Fig. 5C–D).

The GO analysis results were further complemented by GSEA on a whole-genome scale. GSEA based on BP GO annotations showed that the cell cycle process was upregulated in the GM-CSF group (Fig. 5E). Endometrium-related genes were closely depicted in the network diagram (Fig. 5F, G). Notably, Col12a1 encodes type XII collagen α-chain, which is highly expressed in the endometrium, plays a crucial role in embryonic development, and was found to be upregulated in the IUA study that was reported [26], and it was upregulated in the GM-CSF perfusion group.

## Discussion

Patients with a thin endometrium often struggle to achieve a successful pregnancy. Researchers are actively exploring effective methods and medications to increase endometrial thickness and improve endometrial receptivity and the clinical pregnancy rate [9–11]. Studies reported that granulocyte colony-stimulating factor (G-CSF) had a positive effect on endometrial regeneration and increased the number of mesenchymal and hematopoietic stem cells in bone marrow [27, 28]. Recently, several studies have reported improvements in endometrial thickness with the administration of bone marrow mesenchymal stem cells [29, 30]. To verify if the G-CSF family member GM-CSF has the same function as G-CSF, we successfully established a thin endometrium mouse model in this study. A mouse model of thin endometrium was established using 95% ethanol, and then GM-CSF was administered via intrauterine perfusion after two weeks. Finally, we confirmed the therapeutic effect of GM-CSF treatment and explored the possible underlying mechanisms of GM-CSF action.

Ethanol sclerotherapy was previously performed to induce intrauterine adhesions by instilling ethanol into the uterine cavity for over 5 min [21, 31]. 95% ethanol was used to thin the endometrium for less than 1 min [20]. Furthermore, mechanical methods such as scratching the uterine cavity were also applied to mimic a thin endometrium [32]. The mechanical methods require careful and consistent application. Prolonged exposure to ethanol can lead to dehydration and protein denaturation [33]. Therefore, in our study, we used 95% ethanol for only 30 s. Endometrium thickness and uterine morphology were assessed using HE staining, confirming the successful induction of a thin endometrium in mice. Immunohistochemistry revealed a reduction in the number of glands in the thin endometrium of the mouse model. In previous murine experiments, the method of drug release into the uterine cavity was debated. Intraperitoneal injection may not ensure easy drug absorption, while vaginal injection may cause the drug to flow into a side we are not aware of, making it unclear on which side the drug is effective [20]. Furthermore, we used intrauterine injection to simulate human uterine perfusion.

Based on the variation in endometrial thickness and the expression levels of FOXA2 and KI67, our findings indicate that GM-CSF administration effectively enhances endometrial thickness and expedites the regeneration of stromal cells in the thin endometrium mouse model. Previous single-cell RNA sequencing studies also revealed stromal and epithelial cell senescence in thin endometrium [34]. Our animal model also demonstrated this and revealed that GM-CSF improved the embryo implantation site on the thin side of the uterus.

GM-CSF improves the receptivity of the thin endometrium by promoting glandular and stromal cell proliferation in the mouse model. These results were confirmed in the human endometrial cells. GM-CSF had a proliferative effect on endometrial glandular cells but not on stromal cells in humans. This discrepancy may be attributed to differences in physiological positioning within the endometrium [20]. Once the injury signal arrives, the stromal cells may receive it and migrate to the wounded area, thus slowing down the healing process. Additionally, species-specific variations in developmental stages, such as humans being in the proliferative phase and mice in the secretory phase, may also contribute to the differential response.

We further explored the effects of GM-CSF through RNA-seq and found that GM-CSF upregulated genes related to repair and regeneration. Upregulated GO terms related to angiogenesis, cell adhesion, and epithelial cell proliferation were enriched in GM-CSF treatment. The presence of persistent inflammation is an obstacle to tissue healing. Downregulated terms, such as the inflammatory response, can accelerate the resolution of inflammation, leading to quicker healing of injured tissue remodeling [32]. Another report also showed that most genes related to the cell cycle were significantly inhibited in the thin endometrium, which supports our GSEA results that GM-CSF restored the cell cycle process [35].

GM-CSF therapy can increase endometrial thickness and improve the clinical pregnancy rate in thin endometria. The participants in their study were nearly four times more than ours, and their participants were up to 304 [36, 37]. Although there were no significant differences in endometrium thickness, implantation rate, and clinical pregnancy rate were higher in the GM-CSF treatment group compared to those in the saline group in our study. Zhou et al. analyzed the lack of benefits of GM-CSF on embryo quality but found a decrease in the occurrence of chemical pregnancy in women over 35 years old, suggesting that GM-CSF improved implantation competence [38]. However, the delivery rate of GM-CSF did not meet expectations, potentially due to complex factors such as maternal health, nutritional status, and fetal genetics. Another notable finding was the difference in the number of embryos transferred. The embryo transferred on D5 showed better delivery rates and endometrial thickness compared to D3 embryos. As the embryo was cultured for more days, its quality could be tested more easily, which in turn enhances its reliability.

The innovative method in our clinical study involves endometrial scratching before drug perfusion. More studies tend to administer drugs directly to the uterine cavity. Once a slight wound is formed, GM-CSF could increase the secretion of metalloproteinases and promote autolytic debridement by activating local inflammatory cells [37]. The endometrial scratch did not influence the drug treatment of thin endometrium and resulted in a higher clinical pregnancy rate [38]. We speculate that this scratching technique may have also improved the delivery rate in the Saline group.

Though our study demonstrated significant effects of GM-CSF on endometrium thickness and regeneration during proliferation, it was noted that the mouse model may not be sufficiently representative of patients with thin endometrium. Our model may represent only one possible cause of injury induction, but many factors can lead to a thin endometrium, such as Intrauterine adhesions (IUA) and myometriosis [39–41]. Consequently, the relationship between the ethanol mouse model and thin endometrium remains to be further explored in future studies. We speculate that the participants with small sizes in the present study and a larger sample size will be expanded in the subsequent research to achieve a conclusive outcome through multi-center research. We can use multi-omics techniques to further explore the mechanism by which GM-CSF improves endometrial receptivity. GM-CSF can be added to embryo culture before transfer, along with endometrial scratching and intrauterine perfusion, which may help elucidate its role in managing thin endometrium in both experimental and clinical settings.

## Conclusion

In conclusion, our study demonstrated that GM-CSF significantly increases endometrium thickness and gland number, promotes the proliferation of stromal cells, and improves the embryo implantation sites in a mouse model. In human endometrial cells, GM-CSF markedly stimulated the proliferation of glandular epithelial cells, although no significant effect was observed on stromal cells. These findings collectively support the beneficial role of GM-CSF in facilitating endometrial regeneration and improving pregnancy outcomes in the context of thin endometrium pregnancy outcomes in thin endometrium.

## Materials and methods

### Ethical approval

The research was approved by the Ethics Committee of Chongqing Health Center for Women and Children (2018-RGI-1204). Informed written consent was obtained from each participant before they took part in the studies. For animal studies, feeding, disposing, dosing, experimental processes, and sample collection were conducted in accordance with the principles and procedures approved by the Ethics Committee of the laboratory Animal Center of Chongqing Health Center for Women and Children (NO. 2023001). We affirm that all human and animal studies in our experiments were performed in accordance with relevant regulations. The clinical trial registration number (at chictr.org) is 281376.

### Study participants and treatment protocols

All participants included in the study met specific inclusion criteria, which included being 25–40 years old, having experienced failed IVF-ET / FET at least once, having an endometrial thickness of less than 7 mm after a natural cycle or hormone replacement therapy, having a thin endometrium confirmed through repeated examinations, and showing no abnormalities in hysteroscopy within the past 6 months.

The exclusion criteria were diminished ovarian reservation: Anti-müllerian hormone, (AMH) < 1.2 ng/mL, antral follicle counting (AFC) < 7, or basal follicle-stimulating hormone (FSH) > 10 miu/mL, a history of ovariectomy, endometriosis, polycystic ovary syndrome (Rotterdam criteria), and other endocrine disorders, adenomyosis uterine malformation and intramural fibroids larger than 3 cm in diameter. Finally, 84 participants were divided into a control group (Saline, *n* = 38) and an experimental group (GM-CSF, *n* = 46). They were randomly selected and blinded. The endometrium was gently scratched, and endometrial tissues were collected at the same time to be stored frozen and embedded in paraffin after being scratched, and the uterus was infused with Saline or GM-CSF once daily for four days at the end of the first menstrual period. Endometrial scratching and drug infusion were performed in the same way at the end of the second menstrual period. Frozen embryo transplantation was performed during the third menstrual cycle. Endometrial preparation before frozen-thawed embryo transfer was artificial endometrial preparation (AEP). Women received an estradiol (E2) regimen that was delivered orally (4 mg twice a day, Progynova, Bayer, Germany) from Day 2~3 of menstruation and continued for a minimum of 2 weeks, followed by the addition of daily vaginal progesterone gel at a dose of 90 mg per day (Crinone 8% gel, Serono, Germany). Day 3 embryo transfer was performed on the fourth day of progesterone exposure, and Day 5 embryo transfer was performed on the sixth day. The endometrium thickness was recorded on the date of embryo transfer. The pregnancy test was performed at least 14 days after embryo transfer. Upon successful pregnancy, progesterone was continuously applied until 12 weeks of gestation, and the amount of estrogen was gradually reduced.

The main evaluation criterion was confirmed pregnancy as indicated by the presence of a gestational sac detected through vaginal sonography 28–30 days after embryo transfer. The fetal birth determined the final pregnancy evaluation indicator. Secondary evaluation indicators include endometrial thickness, miscarriage rate, and implantation rate, which is determined by the number of live embryos.

### Thin endometrium mice model

A total of 40 6–8-week-old ICR mice were housed in the animal center under controlled conditions and fed with suitable water and food. Superovulation was applied first to ensure all female mice were in estrus synchronization. Mice were injected intraperitoneally with 10 IU of pregnant mare serum gonadotropin (PMSG; Sigma-Aldrich, St. Louis, MO, USA), followed by injection of 10 IU of human chorionic gonadotropin (HCG; Sigma-Aldrich) 48 h later. All mice were operated on three days after HCG injection in diestrus to ensure accurate ethanol injury. 2,2,2-Tribromoethanol (25 g/kg, i.p.) was used to anesthetize mice. All operations were performed with sterile instruments. The mouse model was established to simulate a thin endometrium in humans using chemical injury [20]. For all models, randomization of animals and blinding of examiners were performed. One uterine horn (Thin) was injured by injecting 20 μL 95% ethanol for 30 s and then flushed with 20 μL 0.9% saline. In contrast, the other horn (Sham) was stabbed with a small hole for a sham surgery as a control. The efficacy of the thin endometrium model was evaluated two weeks after surgery.

Mice were randomly apportioned into two groups: the saline group (Saline) and the GM-CSF-treated group (0.2 μg) (GM-CSF). Figs. 1A, 2A, and 3A showed the construction and the treatment process of the thin endometrium mice model in the following brief steps: (1) mouse model construction; (2) uterus collection and drug treatment two weeks later; (3) uterus collection and cohabitation with fertile male mice; (4) uterus collection on pregnant day 3.5; (5) embryo collection on day 7.5. All tissues were collected in a timely manner for later examination.

### H&E staining

H&E staining was used to assess murine endometrium thickness. Collected tissues were embedded in paraffin after being fixed with 4% paraformaldehyde overnight. Then, the waxes were sectioned 5 μm thick to be dyed with hematoxylin and eosin by the standard process. The measurement of endometrium thickness was analyzed using ImageJ.

### Immunohistochemistry

The gland number and the proliferation of endometrial cells were detected by the immunohistochemistry protocol [26]. Briefly, antigen repair was performed in sodium citrate solutions (pH = 6.0) in the microwave oven after dewaxing and rehydration. Endogenous peroxidase was soaked in 3% hydrogen peroxide for 15 min. Then, the slides were incubated with 10% horse serum at 37 °C for one hour. Primary antibody (FOXA2, Abcam, 1:200; KI67, Axl-bio, 1:800) was incubated overnight at 4 °C. Biotin-labeled secondary antibody was used for one hour, and avidin-HRP for 0.5 h at 37 °C. The diaminobenzidine buffer (ORIGENE, Beijing, China) stained positively brown cells, and hematoxylin was counterstained. The sides were dehydrated and coverslip-mounted.

### Fertility assessment in mice

The remaining 20 model mice were mated with fertile males in the same cage with a 1:1 (female: male) ratio two weeks after surgery in the evening. The female mice were examined for vaginal plugs to determine whether they were pregnant or not the following morning. The day of pregnancy was defined as E0.5. Uteri were collected to detect the proliferation of the endometrium on E3.5. The number of embryo implantation sites and the weight of embryos were collected on E7.5 to evaluate fertility.

### Library preparation and transcriptome sequencing

Human endometrial tissues were selected randomly for RNA-seq. Briefly, total RNA extraction and library preparation were carried out according to the manufacturer’s instructions. Poly-T oligo-attached magnetic beads were used for mRNA purification. Fragmentation was performed by divalent cations in the First Stand Synthesis Reaction Buffer (5×). First-strand cDNA was synthesized with M-MuLV reverse transcriptase and random hexamer primer. Second-strand cDNA was synthesized subsequently using DNA polymerase I and RNase H. Adaptors with hairpin loop structures were used to ligate for hybridization after adenylation of the 3′ ends of the DNA fragments. The library fragments were purified with VAHTS DNA Clean Beads (Vazyme, Nanjing, China). PCR was amplified with Phusion High-Fidelity DNA polymerase, Index primer, and universal primers. PCR products were purified, and library quality was finally checked with the Agilent Bioanalyzer 2100 system. Dual-indexed libraries were pooled and sequenced in paired-end reads on the Novaseq platform (Illumina).

### RNA-seq data analysis

According to our previous study, clean data were obtained by removing reads containing adapters and low-quality reads from raw data by HISAT2 (version 2.1.0) [42]. The read count of genes based on the annotation file was gained by FeatureCounts (version 2.0.1). The analysis of differential gene expression was carried out using the DESeq2 (version 1.30.0) R package. Those genes with significant changes (absolute log2 fold change > 1 and *p*-value < 0.05) were considered differentially expressed genes (DEGs).

Gene Set Enrichment Analysis (GSEA) was performed with the GSEA software tool (version 4.1.0, http://www.gsea-msigdb.org/gsea/downloads.jsp). Gene Ontology (GO) analysis and network analysis were used in the Metascape tool and the String tool, respectively.

### Statistical analysis

All data were analyzed using GraphPad Prism, ver. 8.0 software and presented as mean ± SEM. The mouse results were analyzed by a two-tailed Student’s *t*-test or two-way analysis of variance (ANOVA) with multiple comparisons to determine significance in two-group and multiple-group experiments. Most of the clinical data were summarized as mean ± SEM for continuous data and number (percentage) for categorical data by groups with a two-tailed Student’s *t*-test. The data meet the assumptions of the test's normal distribution. Implantation and pregnancy outcomes were used in the χ^2^ test. *P*-value < 0.05 was considered statistically significant. There is an estimate of variation within each group of data, and the variance is similar between the groups that are being statistically compared.

## Supplementary information

Supplementary information

## Data Availability

The datasets used and/or analyzed during the current study are available from the corresponding author upon reasonable request. The raw sequence data reported in this paper have been deposited into the Genome Sequence Archive for human: HRA010883.
