# Supplementary material for: GM-CSF improves the receptivity of thin endometrium by promoting glandular and stromal cell proliferation in mice and humans
Source: Cell Death Discov. 2025 Dec 29;12:65. doi: 10.1038/s41420-025-02928-5 (PMC12848295; doi:10.1038/s41420-025-02928-5)
Supplement: Supplementary file 1 — Supplementary information [file 41420_2025_2928_MOESM1_ESM.docx]

**Supplementary tables**

**Table S1** Baseline patients' characteristics for the embryo transferred on D3.

|  | GM-CSF  （n=29） | Saline  （n=25） | *P* value |
| --- | --- | --- | --- |
| Age (years) | 34.21 ± 4.69 | 34.08 ± 3.25 | 0.910 |
| Infertility duration  (years) | 4.31 ± 2.47 | 6.77 ± 4.07 | 0.055 |
| BMI (kg/m^2^) | 22.57 ± 2.61 | 21.54 ± 2.47 | 0.145 |
| AMH (ng/ml) | 3.89 ± 3.01 | 3.67 ± 2.37 | 0.774 |
| Basal FSH (IU/L) | 5.84 ± 1.80 | 5.92 ± 1.85 | 0.876 |
| Basal LH (IU/L) | 3.46 ± 1.48 | 3.63 ± 1.90 | 0.720 |
| Basal E2 (IU/L) | 35.41 ± 1.48 | 32.22 ± 10.70 | 0.400 |
| Basal PGR (IU/L) | 0.30 ± 0.14 | 0.33 ± 0.16 | 0.450 |
| Previous IVF failure (n) | 2.00 ± 1.20 | 1.64 ± 1.50 | 0.330 |
| Case of infertility |  |  |  |
| Tubal factor | 15 (51.72%) | 17 (68%) | 0.225 |
| Other female factors | 2 (6.90%) | 1 (4%) | 0.643 |
| Mixed factors | 12 (41.38%) | 6 (24%) | 0.177 |
| Unknown factors | 0 | 1 (4%) | 0.277 |

Abbreviations: BMI, body mass index; AMH, anti-Müllerian hormone; FSH, follicle-stimulating hormone; LH, luteinizing hormone; E2, estradiol; PGR, progesterone receptor; IVF, in vitro fertilization; GM-CSF, granulocyte macrophage colony-stimulating factor. Values are given as mean ± SD or number (percentage).

**Table S2.** Pregnancy outcomes of the embryo transferred on D3.

|  | GM-CSF  （n=29） | Saline  （n=25） | *P* value |
| --- | --- | --- | --- |
| Embryo transfer (n) | 55 | 50 | - |
| Implantation rate (%) | 11/55 (20%) | 7/50 (14%) | 0.415 |
| Clinical pregnancy rate | 10/29 (34.48%) | 5/25 (20%) | 0.236 |
| Singleton pregnancy rate | 10/29 (34.48%) | 4/25 (16%) | 0.143 |
| Multiple pregnancy rate | 0/29 (0%) | 1/25 (4%) | 0.143 |
| Abortion rate | 5/10 (50%) | 0/5 (0%) | 0.053 |
| Delivery rate | 5/10 (50%) | 5/5(100%) | 0.053 |

Values are given as a number (percentage).

Abortion rate indicates the number of pregnant women giving misbirth / clinical pregnancy.

Delivery rate shows the number of pregnant women giving live birth / clinical pregnancy.

**Table S3.** The endometrium thickness of the embryo transferred on D3.

|  | GM-CSF  （n=29） | Saline  （n=25） | *P* value |
| --- | --- | --- | --- |
| Endometrium thickness in previous cycle (mm) | 5.97 ± 0.78 | 5.92 ± 0.95 | 0.848 |
| Endometrium thickness in the implanted cycle (mm) | 6.03 ± 0.73 | 6.00 ± 0.50 | 0.843 |

Values are given as mean ± SD by group.

**Table S4.** Baseline patient characteristics of the embryo transferred on D5.

|  | GM-CSF  （n=16） | Saline  （n=13） | *P* value |
| --- | --- | --- | --- |
| Age (years) | 31.44 ± 3.90 | 32.54 ± 2.93 | 0.407 |
| Infertility duration  (years) | 4.31 ± 2.47 | 6.77 ± 4.07 | 0.055 |
| BMI (kg/m^2^) | 23.31± 6.26 | 22.85 ± 2.28 | 0.803 |
| AMH (ng/ml) | 6.15 ± 4.26 | 3.87 ± 2.13 | 0.091 |
| Basal FSH (IU/L) | 5.10 ± 1.55 | 4.63 ± 1.16 | 0.365 |
| Basal LH (IU/L) | 3.61 ± 2.16 | 3.10 ± 1.99 | 0.522 |
| Basal E2 (IU/L) | 33.84 ± 21.67 | 38.14 ± 17.08 | 0.565 |
| Basal PGR (IU/L) | 0.22 ± 0.15 | 0.27 ± 0.14 | 0.374 |
| Previous IVF failure (n) | 1.56 ± 0.73 | 1.08 ± 0.28 | 0.031 |
| Case of infertility |  |  |  |
| Tubal factor | 7 (43.75%) | 6 (46.15%) | 0.897 |
| Other female factors | 3 (18.75%) | 3 (23.08%) | 0.775 |
| Mixed factors | 6 (37.50%) | 4 (30.77%) | 0.705 |
| Unknown factors | 0 | 0 | - |

Abbreviations: BMI, body mass index; AMH, anti-Müllerian hormone; FSH, follicle-stimulating hormone; LH, luteinizing hormone; E2, estradiol; PGR, progesterone receptor; IVF, in vitro fertilization; GM-CSF, granulocyte macrophage colony-stimulating factor. Values are given as mean ± SD or number (percentage).

**Table S5.** Pregnancy outcomes of embryo transferred on D5.

|  | GM-CSF  （n=16） | Saline  （n=13） | *P* value |
| --- | --- | --- | --- |
| Embryo transfer (n) | 26 | 19 | - |
| Implantation rate (%) | 7/26 (26.92%) | 5/19 (26.32%) | 0.964 |
| Clinical pregnancy rate | 5/16 (31.25%) | 5/13 (38.46%) | 0.387 |
| Singleton pregnancy rate | 3/16 (18.75%) | 5/13 (38.46%) | 0.114 |
| Multiple pregnancy rate | 2/16 (12.50%) | 0/13 (0%) | 0.114 |
| Abortion rate | 0/5 (0%) | 1/5 (20%) | 0.292 |
| Delivery rate | 5/5 (100%) | 4/5 (80%) | 0.292 |

Values are given as a number (percentage).

Abortion rate indicates the number of pregnant women giving misbirth / clinical pregnancy.

Delivery rate shows the number of pregnant women giving live birth / clinical pregnancy.

**Table S6.** Endometrium thickness of embryo transferred on D5.

|  | GM-CSF  （n=16） | Saline  （n=13） | *P* value |
| --- | --- | --- | --- |
| Endometrium thickness in previous cycle (mm) | 5.56 ± 0.51 | 5.65 ± 0.47 | 0.626 |
| Endometrium thickness in the implanted cycle (mm) | 6.25 ± 0.77 | 6.23 ± 1.17 | 0.958 |

Values are given as mean ± SD by group.
